# Expanding the theory of planned behavior to predict fruits and vegetables consumption among a sample of women in Saudi Arabia

**DOI:** 10.3389/fpubh.2025.1720598

**Published:** 2025-12-04

**Authors:** Howeida Abusalih, Sarah A. Alasmari, Suha Hashim Abduljawad, Ameerah Almaski, Manal Almughamisi, Asmaa Fatani, Bayan Tashkandi, Abeer A. Aljehani, Afnan H. Saaty, Nada Benajiba

**Affiliations:** 1 Department of Health Sciences, College of Health and Rehabilitation Sciences, Princess Nourah bint Abdulrahman University, Riyadh, Saudi Arabia; 2 College of Health Sciences, Public Health Department, Saudi Electronic University, Riyadh, Saudi Arabia; 3 College of Applied Medical Sciences, Department of Clinical Nutrition, Taibah University, Medina, Saudi Arabia; 4 Food and Nutrition Department, Human Sciences and Design Faculty, King Abdulaziz University, Jeddah, Saudi Arabia; 5 Ibn Tofail University-CNESTEN, Joint Research Unit in Nutrition and Food, RDC-Nutrition AFRA/IAEA, Rabat, Morocco

**Keywords:** fruits and vegetables consumption, adult women, perceived behavioral control, intention, social norms, attitude, theory of planned behavior, Saudi Arabia

## Abstract

**Background:**

Adequate fruit and vegetable (F&V) intake is a key component of a healthy diet and is influenced by both psychological and environmental factors.

**Aim:**

to explore determinants of F&V consumption among Saudi adult women, applying an extended Theory of Planned Behavior (TPB) model.

**Methods:**

A cross-sectional survey recruited 476 Saudi adult women aged 18–59 across all five regions of Saudi Arabia. Data were collected using a validated online questionnaire assessing TPB constructs, socio-demographics, lifestyle behaviors, and dietary patterns. Structural equation modeling (SEM) was used to test both the original and extended TPB models.

**Results:**

Participants held positive attitudes but demonstrated low adherence, consuming a mean of 3.33 F&V servings daily. In the original TPB model, intention, and perceived behavioral control (PBC) significantly predicted behavior (R^2^ = 31%), while attitude, subjective norms, and PBC predicted intention (R^2^ = 75%). The extended model explained 45% of the variance in behavior, with PBC as the sole direct significant predictor. Knowledge and family meal frequency positively influenced attitudes, while F&V purchasing, and a healthy diet were associated with higher PBC.

**Conclusion:**

PBC emerged as the only direct predictor of behavior in the extended model. Moreover, this study highlighted the crucial role of PBC in translating intention into the actual consumption of fruits and vegetables among Saudi women. Family-centered strategies that strengthen women's confidence and skills in healthy meal planning may effectively bridge the intention–behavior gap.

## 1 Introduction

Healthy dietary behaviors are consistent eating patterns that promote physical and mental wellbeing, reduce the risk of chronic diseases, and support healthy growth and development throughout life (1–3). These behaviors include a wide range of practices, including meal regularity (4), moderation in portion sizes (5), balanced nutrient intake, and adherence to dietary guidelines (2). They are shaped by a complex interplay of factors, such as individual attitudes, health literacy, cultural traditions, family and social influences, food environment, and socioeconomic determinants (6, 7).

Among the most important components of healthy dietary behaviors is the regular consumption of fruits and vegetables (F&V) (2). Fruits and vegetables are rich sources of dietary fiber, vitamins, minerals, and bioactive compounds that provide protective effects against cardiovascular disease, type 2 diabetes, obesity, and certain cancers (8, 9). Evidence from both epidemiological and intervention studies indicates that individuals who meet recommended F&V intake —approximately 400 grams per day, equivalent to five servings of about 80 grams each—have lower all-cause mortality and reduced risk of noncommunicable diseases (10).

Despite their proven benefits, global F&V consumption remains below recommended levels globally (11). In Saudi Arabia, as in many Middle Eastern contexts, studies have shown inadequate daily intake of F&V, with cultural habits, knowledge gaps, and environmental factors acting as major barriers (12, 13). Researchers frequently employ psychological models to investigate and promote improved dietary practices, with the Theory of Planned Behavior (TPB) being a prominent example (14). This framework posits that an individual's actions are primarily determined by three fundamental components: attitude (one's favorable or unfavorable appraisal of a behavior), subjective norms (the perceived expectations from one's social circle), and perceived behavioral control (PBC), which concerns the perceived feasibility of executing the behavior (14). The applicability of the TPB for forecasting and dissecting various health-related conducts, such as food choices, has been validated in numerous cultural environments (15–17). Its reliability in clarifying intentions related to diet, especially concerning the intake of F&V intake), is further corroborated by contemporary meta-analyses (25, 54). Evidence indicates that the model's components account for a substantial amount of the variation in people's plans to eat F&V, as well as their real consumption habits (16). However, beyond the core elements of the TPB, research also highlights the significant influence of sociodemographic and lifestyle variables on eating patterns. Factors including age, gender, educational attainment, income, understanding of nutrition, familial routines, and levels of physical activity have all been recognized as important determinants (18–22).

**Figure 1:**
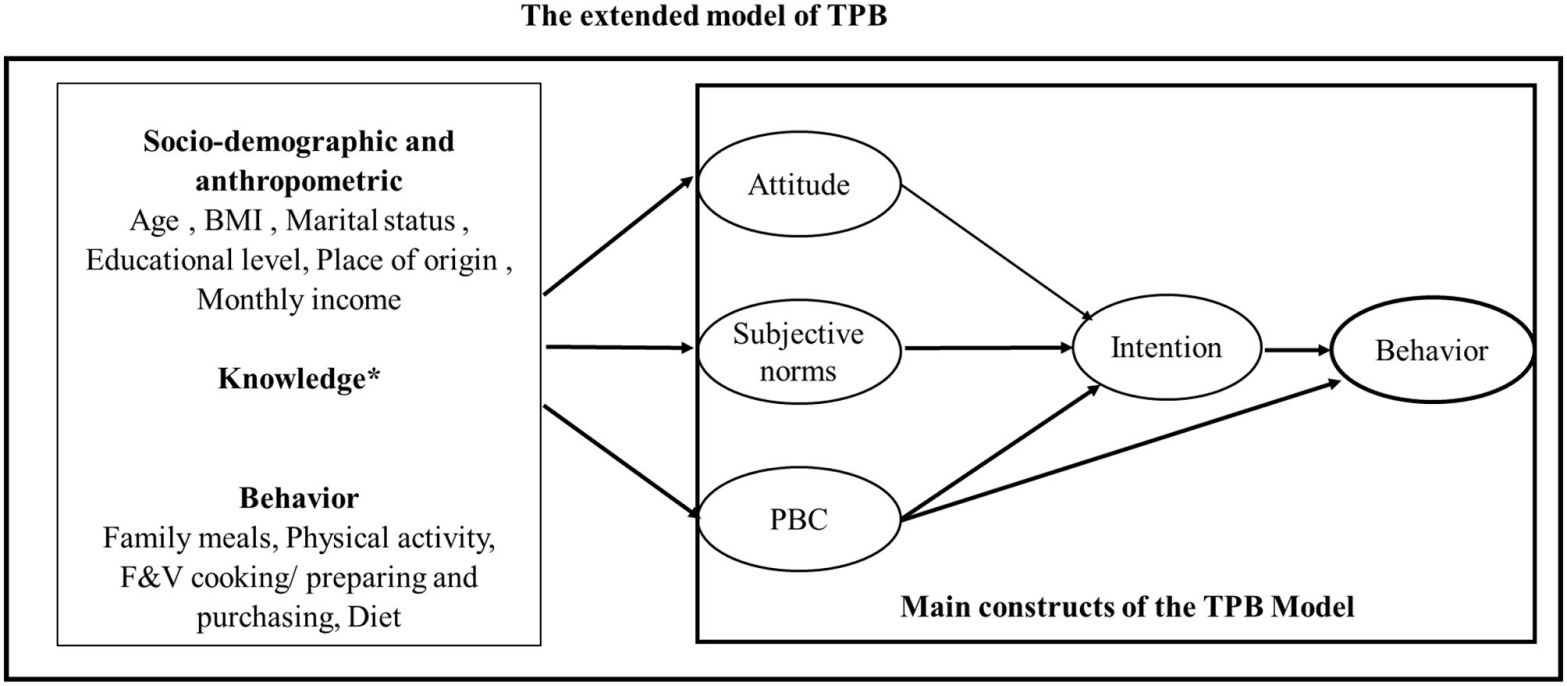
TPB-extended model. *Knowledge regarding the WHO dietary consumption recommendations.

Given the importance of F&V consumption as a cornerstone of healthy dietary behavior and the persistent gap between recommendations and actual intake, especially in Saudi Arabia, it is essential to investigate the psychosocial and contextual determinants of this dietary behavior. The present study applies the TPB extended model (Figure 1) to identify factors influencing F&V consumption among adult women in Saudi Arabia, to provide a ground foundation for developing culturally adapted nutrition promotion strategies.

## 2 Methods

### 2.1 Study population and design

This research study used a cross-sectional design and was conducted across the five regions of Saudi Arabia: Northern, Southern, Eastern, Western, and Central. Data collection was performed from May to October 2024. Eligible participants were Saudi nationals living in the country during the study period, aged 18–59 years. Individuals aged ≥60 years old were excluded due to well-documented physical, mental, and behavioral changes affecting their dietary choices and health outcomes (23). Moreover, older adults may represent a population for which the predictive power of the TPB is diminished, because of their reliance on habitual behaviors and environmental cues over deliberate intention in dietary choices (24, 25). The study exclusion criteria were pregnant and lactating women, individuals following prescribed or restrictive diets, and patients with chronic conditions influencing fruit and vegetable consumption. Ethical approval for the study was obtained from the Institutional Review Board (IRB) at King Abdulaziz University (Log number 15–24).

### 2.2 Sampling approach and sample size estimation

A non-probability, convenience sampling strategy was employed to collect online data. In the absence of national statistics on the proportion of Saudi adults meeting the recommended daily intake of five portions of fruits and vegetables, a prevalence rate of 50% was assumed. This conservative estimate is standard in epidemiological studies when prior data are unavailable, as it ensures the largest possible sample size and maximizes statistical precision (26). The sample size calculation utilized a standard formula, anchored to an adult population of approximately 13 million in Saudi Arabia (57).

n = Z^2^ × p (1 – p)/d^2^, where:

- *n* = required sample size
- *Z* = Z-score corresponding to the desired confidence level (1.96 for 95%)
- *p* = estimated proportion (set at 0.50 to maximize sample size)
- *d* = margin of error (set at 0.05)

The calculation indicated a minimum of 385 participants. To allow for incomplete or invalid responses, an additional 10% was added, bringing the required sample size to 422. Ultimately, the study recruited 467 valid participants.

### 2.3 Data collection procedure

The research tool was an anonymous online survey using Google Forms. Participants were recruited through an official email from the University's Scientific Research Committee to staff and students. Then, a snowball sampling method was used, asking recipients of the email to share the survey on their usual social media platforms. The first page of the survey required informed consent, explaining the study's goals, voluntary participation, and the right to withdraw. Only those who provided consent could access the questionnaire. To ensure clarity and reduce response bias, the survey was designed in Arabic, the participants' native language. No personal identifiers were collected to maintain data security and confidentiality.

### 2.4 Data collection instrument

A structured questionnaire was used, organized into four main sections:

- **Section 1—Sociodemographic and anthropometric data**: Age, gender, marital status, education, region of residence, and monthly household income in Saudi Riyals (SAR). Height and weight were also collected to calculate body mass index (BMI, kg/m^2^).
- **Section 2—Nutrition knowledge**: One item assessed participants' awareness of the **(author?)** (56) recommendation of consuming five daily servings of fruits and vegetables.
- **Section 3—Lifestyle behaviors**: Questions covered physical activity, family meal frequency, type of diet (healthy, balanced, or calorie-focused), and involvement in purchasing and preparing fruits and vegetables (22).
- **Section 4—TPB constructs**: Based on Ajzen's [(14, 55)] framework and the research study by Menozzi et al. (22), the Target, Action, Context, and Time (TACT) principle defined the behavior of interest as “consuming five servings of fruits and vegetables daily over the next week.” Attitudes were measured with four semantic differential items (e.g., healthy–unhealthy, pleasant–unpleasant). SN were assessed with two items regarding family and friends' expectations. PBC was measured with two items on perceived ability and confidence. Intention was evaluated using three items reflecting participants' goals and likelihood of meeting the F&V target. Behavior was assessed with two questions asking about actual servings consumed and the number of days participants achieved the target during the previous week. All items were rated on a five-point Likert scale as follows: strongly disagree = 1, disagree = 2, neutral = 3, agree = 4, and strongly agree =5. The sum of scores obtained by each participant for all items under each construct was used to generate composite scores for that construct.

### 2.5 Validity testing and reliability

The finalized questionnaire was reviewed by 11 experts in nutrition, psychology, and public health to assess face and content validity, following established guidelines (27, 28). Experts evaluated clarity, comprehensiveness, and completion time, with 88.9% rating the content as comprehensive and easy to understand. The average completion time was 8.9 ± 1.4 min. Content validity indices showed strong agreement: item-level CVI (I-CVI) ranged from 0.82 to 1.00, the scale-level CVI/Average was 93.3%, and the scale-level CVI/Universal Agreement was 77.4%. These results confirm that the instrument was conceptually robust and user-friendly (29, 30).

To evaluate reliability, the internal consistency was assessed using Cronbach's alpha. The analysis was conducted with participants who represented the target study population. The internal consistency of all constructs was acceptable to excellent, with Cronbach's alpha ranging from 0.72 to 0.92. (Supplementary Table 1) (31).

### 2.6 Statistical analysis

All statistical analyses were conducted using R software. Descriptive data were summarized as means with standard deviations for continuous variables and as frequencies with percentages for categorical variables. To examine the research hypothesis, structural equation modeling (SEM) was applied. Model fit was evaluated using multiple indices, including chi-square (χ^2^), the Comparative Fit Index (CFI), Tucker–Lewis Index (TLI), Root Mean Square Error of Approximation (RMSEA), and Standardized Root Mean Square Residual (SRMR). The coefficient of determination (R^2^) was reported to indicate the proportion of variance explained in the endogenous variables (intention and behavior). Model adequacy was defined by CFI and TLI values >0.90 and RMSEA and SRMR values below 0.08. All models were estimated using the Maximum Likelihood (ML) method. The extended TPB model incorporated background factors as predictors of attitudes, SN, and PBC. Additionally, correlations between socio-demographic characteristics, nutrition knowledge, and lifestyle behaviors were analyzed independently of the SEM.

## 3 Results

### 3.1 General information of the studied population

A total of 467 Saudi women participated in the study (Table 1, with an average age of 35.5 ± 13.2 years. Nearly half of the sample (46.1%) had a normal BMI (18.5–24.9 kg/m^2^), while an almost equal proportion (46.0%) was either overweight or obese. Half of the participants (49.9%) were married, and 50.1% unmarried. Regarding education, over half (54.9%) held a bachelor's degree or diploma, and 36.7% had a postgraduate degree. Most participants were from the Western region (59.3%), followed by the Middle region (30.2%). More than one-third (36.0%) of the participants reported a monthly income of 11,000–20,000 SAR. Knowledge of the WHO daily F&V recommendations was limited. About 29.7% thought fewer than three servings were enough, and 37.7% claimed three servings; only 17.3% and 4.1% recognized five or more than six servings, respectively. Regarding household behaviors, more than half of participants (51.4%) had family meals every day, and 35.6% reported always purchasing F&V. Additionally, 36.4% always prepared or cooked them at home. Most participants considered their diet as neutral in healthiness (45.8%) and neutral in caloric content (48.8%); 42.8% described it as balanced. Physical activity levels were generally low. More than half (59.7%) reported never engaging in intense activity. Furthermore, 37.9 and 33.0% reported no moderate or light activity, respectively. For overall activity that raised their heart rate, one-third (32.8%) reported never being active, while 28.5% stated they only rarely engage in such physical activity.

**Table 1:** Sociodemographic characteristics.

**Parameter**	**Total (*N* = 467)**
**Age**	35.48 ± 13.24
**BMI (Kg/m** ^2^ **)**	
< 18.5	37 (7.9%)
18.5–24.9	215 (46.1%)
25–29.9	107 (22.9%)
>30	108 (23.1%)
**Marital status**	
Married	233 (49.9%)
Not married	234 (50.1%)
**Educational level**	
Secondary/higher	40 (8.4%)
Bachelor/diploma	256 (54.9%)
Postgraduate	171 (36.7%)
**Place origin**	
Northern	7 (1.5%)
Southern	16 (3.4%)
Middle	141 (30.2%)
Eastern	26 (5.6%)
Western	277 (59.3%)
**Monthly income (Saudi Riyals)**	
5,000	51 (10.9%)
5,000–10,000	137 (29.2%)
11,000–20,000	168 (36.0%)
>20,000	111 (23.9%)
**Knowledge regarding the WHO daily dietary consumption**	
**recommendations**	
< 3 servings	139 (29.7%)
3 servings	176 (37.7%)
4 servings	52 (11.2%)
5 servings	81 (17.3%)
≥6 servings	19 (4.1%)
**Family meals**	
Never	19 (4.1%)
1–2/day	79 (16.9%)
3–4/day	75 (16.0%)
5–6/day	54 (11.6%)
Daily	240 (51.4%)
**Fruits and vegetables purchasing**	
Never	14 (3.0%)
Rare	41 (8.7%)
Sometimes	105 (22.6%)
Often	141 (30.1%)
Always	166 (35.6%)
**Fruits and vegetables cooking/preparing**	
Never	27 (5.9%)
Rare	40 (8.4%)
Sometimes	95 (20.4%)
Often	135 (28.9%)
Always	170 (36.4%)
**How healthy the diet is**	
Very unhealthy	9 (1.9%)
Unhealthy	54 (11.6%)
Neutral	214 (45.8%)
Healthy	173 (37.1%)
Very healthy	17 (3.6%)
**How balanced the diet is**	
Very unbalanced	10 (2.2%)
Unbalanced	90 (19.2%)
Neutral	153 (32.8%)
Balanced	200 (42.8%)
Very balanced	14 (3.0%)
**How caloric the diet is**	
Very low caloric	8 (1.7%)
Low caloric	67 (14.3%)
Neutral	228 (48.8%)
High caloric	134 (28.7%)
Very high caloric	30 (6.5%)
**Intense physical activity**	
Never	279 (59.7%)
1 time/month	5 (1.1%)
1 time/week	45 (9.7%)
2–3 times/week	92 (19.7%)
4–6 times/week	30 (6.4%)
At least 1 time/day	16 (3.4%)
**Moderate physical activity**	
Never	177 (37.9%)
1 time/month	4 (0.9%)
1 time/week	66 (14.1%)
2–3 times/week	109 (23.3%)
4–6 times/week	63 (13.5%)
At least 1 time/day	48 (10.3%)
**Light physical activity**	
Never	154 (33.0%)
1 time/month	6 (1.3%)
1 time/week	60 (12.9%)
2–3 times/week	108 (23.1%)
4–6 times/week	66 (14.1%)
At least 1 time/day	73 (15.6%)
**Any type of physical activity leading to increased heartbeats**	
Never	153 (32.8%)
Rare	133 (28.5%)
Sometimes	114 (24.3%)
Often	55 (11.8%)
Always	12 (2.6%)

Table 2 presents the Cronbach's alpha, mean scores, and standard deviations for the constructs. The attitude scale showed generally positive scores on fruit and vegetable consumption, with mean scores ranging from 3.98 ± 0.86 (“not pleasant/pleasant”) to 4.35 ± 0.75 (“unhealthy/healthy”). The SN construct demonstrated that family expectations (3.57 ± 1.03) were slightly higher than friends' expectations (3.37 ± 1.00) regarding eating five servings per day. In terms of PBC, participants reported that eating five servings daily was possible (3.62 ± 0.97) and that they were confident they could achieve it (3.72 ± 0.91). Participants showed moderate intentions, with mean scores of 3.63 ± 0.99 (“I intend”), 3.36 ± 1.01 (“I am sure”), and 3.60 ± 1.03 (“My aim”). Finally, as for the behavior, te average reported number of daily servings in the past week was 3.33 ± 0.90. The frequency of meeting the five-a-day recommendation was relatively low (2.52 ± 1.26), averaging 1–2 days per week.

**Table 2:** Constructs, mean scores, and standard deviations.

**Construct items**	**Mean ±SD**
**Attitude**	
Eating five servings of vegetables and fruits per day next week is:	
Bad/good	4.26 ± 0.81
Not pleasant/pleasant	3.98 ± 0.86
Unhealthy/healthy	4.35 ± 0.75
Indigestible/digestible	4.10 ± 0.83
**Subjective norm**	
My family expects me to eat five servings of vegetables and fruits per day next week	3.57 ± 1.03
My friends expect me to eat five servings of vegetables and fruits per day next week	3.37 ± 1.00
**Perceived behavioral control**	
I think that eating five servings of vegetables and fruits per day next week is possible	3.62 ± 0.97
I am sure I can eat five servings of vegetables and fruits per day next week	3.72 ± 0.91
**Intention**	
I intend to eat five servings of vegetables and fruits per day next week	3.63 ± 0.99
I am sure to eat five servings of vegetables and fruits per day next week	3.36 ± 1.01
My aim is to eat five servings of vegetables and fruits per day next week	3.60 ± 1.03
**Behavior**	
Number of servings daily last week	3.33 ± 0.90
Frequency of consumption	2.52 ± 1.26

The frequency of consumption is measured by the following item: How many days have been eaten five servings of vegetables and fruits last week: 1 = I didn't eat, 2 = 1-2 days, 3 = 3–4 days, 4 = 5–6 days, and 5 = daily.

### 3.2 Application of the TPB model to predict F&V consumption

The structural equation modeling results based on the TPB are shown in Table 3. The model explained 31% of the variance in behavior (R^2^ = 0.31) and 75% of the variance in intention (R^2^ = 0.75). For predictors of F&V consumption behavior, both intention (coeff = 0.19, se = 0.07, *p* = 0.005,) and PBC (coeff = 0.31, se = 0.14, *p* = 0.032) were important contributors. When exploring the intention to consume F&V, all three TPB constructs showed significant relationships. Specifically, attitude (coeff = 0.32, se = 0.12, *p* = 0.008), SN (coeff = 0.36, se = 0.12, p = 0.003), and PBC (coeff = 1.31, se = 0.20, *p* < 0.001) were positively related to intention, with PBC being the strongest predictor. The covariance analysis showed significant positive correlations among the three factors influencing intention. Attitude was strongly correlated with both SN (coeff = 0.56, se = 0.05, *p* = 0.000) and PBC (coeff = 053, se = 0.06, *p* = 0.000). SN was moderately correlated with PBC (coeff = 0.46, se = 0.06, *p* = 0.000).

**Table 3:** TPB model unstandardized coefficients (coeff), standard error (se), standardized coefficients (std), and *p*-values.

**Endogenous variables**	**R^2^**	**coeff**	**se**	**std**	***P***
Behavior:	0.31				
Intention		0.19	0.07	0.32	0.005
Perceived behavioral control		0.31	0.14	0.26	0.032
Intention:	0.75				
Attitude		0.32	0.12	0.16	0.008
Subjective norm		0.36	0.12	0.18	0.003
Perceived behavioral control		1.31	0.20	0.66	0.000
**Covariances and correlations**		**coeff**	**se**	**std**	***P***
Attitude ↔ subjective norm		0.56	0.05	0.56	0.000
PBC↔ subjective norm		0.46	0.06	0.46	0.000
PBC ↔ attitude		0.53	0.05	0.53	0.000

Model fit measures: χ^2^_(57)_ = 115.758; CFI = 0.975; TLI = 0.966; RMSEA (95% CI) = 0.047 (0.036–0.058); SRMR = 0.040.

**Figure 2:**
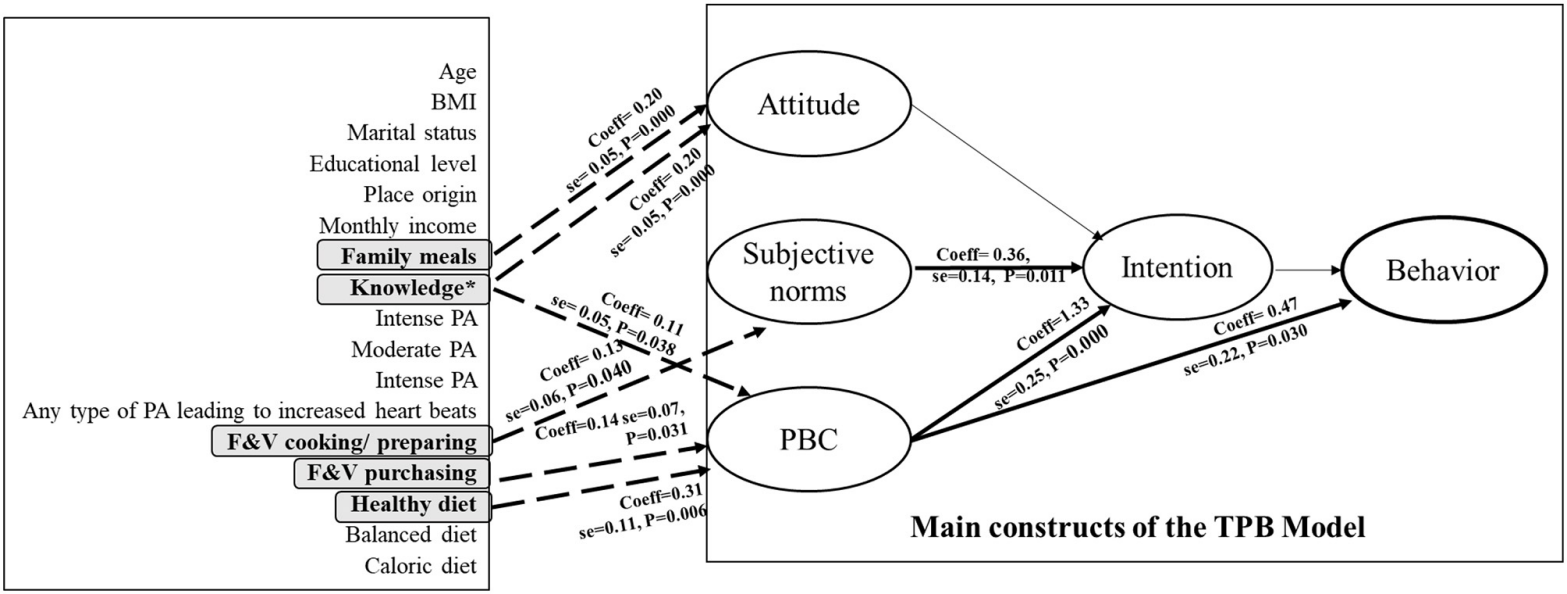
TPB-extended model and significant predictions. BMI, body mass index; PA, physical activity; F&V, fruits and vegetables. *Knowledge regarding the WHO dietary consumption recommendations.

The extended TPB model (Table 4) demonstrated good fit indices [χ^2^_(227_) = 424.73, CFI = 0.952, TLI = 0.909, RMSEA = 0.047 (95% CI: 0.040, 0.054), SRMR = 0.046], indicating an acceptable model fit. The model explained 78% of the variance in intention, 45% in behavior, 21% in attitude, 12% in SN, and 24% in PBC. For behavior prediction, PBC was the only significant predictor (coeff = 0.43, se = 0.22, *p* = 0.030). Intention did not have a significant direct effect (p = 0.338). Regarding intention, both SN (coeff = 0.18, se = 0.14, *p* = 0.011) and PBC (coeff = 0.72, se = 0.25, *p* < 0.001) were significant predictors, while attitude was not statistically significant (*p* = 0.113). Among the background variables, several factors were associated with TPB constructs. Knowledge of WHO recommendations (coeff = 0.29, se = 0.05, *p* = 0.000) and the frequency of family meals (coeff = 0.20, se = 0.05, *p* =0.000) were positively related to attitude. For SN, higher F&V cooking and preparing (coeff = 0.13, se = 0.06, *p* = 0.040) was the only significant contributor. PBC was positively associated knowledge (coeff = 0.11, se = 0.05, *p* = 0.038), F&V purchasing (coeff = 0.14, se = 0.07, *p* = 0.031), and healthy diet (coeff = 0.31, se = 0.11, *p* = 0.006). Sociodemographic factors like age, BMI, marital status, education, place of origin, and income were not significant predictors of any TPB constructs in this model (Figure 2).

**Table 4:** TPB-extended model unstandardized coefficients (coeff), standard error (se), standardized coefficients (std), and *p*-values.

**R^2^**	**Intention**				**Behavior**				**Attitudes**				**SN**				**PBC**			
	**0.78**				**0.45**				**0.21**				**0.12**				**0.24**			
	**coeff**	**se**	**std**	***P***	**coeff**	**se**	**std**	***P***	**coeff**	**se**	**std**	***P***	**coeff**	**se**	**std**	***P***	**coeff**	**se**	**std**	***P***
Attitude	0.21	0.13	0.11	0.113																
SN	0.36	0.14	0.18	**0.011**																
PBC	1.33	0.25	0.72	**0.000**	0.47	0.22	0.43	**0.030**												
Intention					0.10	0.11	0.17	0.338												
Age									−0.01	0.01	−0.08	0.310	0.01	0.01	0.12	0.181	−0.00	0.01	−0.01	0.853
BMI									0.01	0.07	0.01	0.197	−0.03	0.07	−0.02	0.725	0.07	0.07	0.06	0.317
Marital status									0.12	0.16	0.05	0.438	0.09	0.16	0.04	0.546	0.14	0.17	0.06	0.409
Educational level									0.04	0.09	0.02	0.668	−0.05	0.08	−0.03	0.576	−0.04	0.09	−0.02	0.705
Place origin									−0.00	0.06	−0.00	0.985	−0.05	0.06	−0.05	0.416	−0.04	0.05	−0.04	0.455
Monthly income									0.02	0.08	0.02	0.768	−0.07	0.07	−0.06	0.303	−0.04	0.07	−0.03	0.609
Knowledge^*^									0.29	0.05	0.31	**0.000**	0.09	0.05	0.10	0.097	0.11	0.05	0.12	**0.038**
Family meals									0.20	0.05	0.23	**0.000**	0.07	0.05	0.09	0.154	0.06	0.05	0.07	0.202
F&V purchasing									0.05	0.07	0.04	0.473	−0.05	0.06	−0.05	0.417	0.14	0.07	0.13	**0.031**
F&V cooking/ preparing									0.00	0.06	0.00	0.997	0.13	0.06	0.15	**0.040**	0.08	0.06	0.08	0.196
Healthy diet									0.15	0.11	0.11	0.155	0.06	0.11	0.05	0.579	0.31	0.11	0.22	**0.006**
Balanced diet									−0.10	0.10	−0.08	0.318	−0.01	0.11	−0.01	0.949	0.01	0.09	0.01	0.896
Caloric diet									0.13	0.08	0.10	0.092	0.09	0.07	0.07	0.215	0.03	0.07	0.02	0.706
Intense PA									−0.02	0.05	−0.03	0.655	0.07	0.04	0.11	0.090	0.02	0.04	0.03	0.567
Moderate PA									−0.00	0.04	−0.01	0.925	0.06	0.04	0.10	0.129	0.07	0.04	0.11	0.075
Light PA									0.01	0.03	0.01	0.823	0.01	0.03	0.02	0.697	0.04	0.03	0.07	0.187
Any type of PA leading to increased heartbeats									−0.02	0.08	−0.02	0.803	−0.11	0.07	−0.11	0.140	0.02	0.07	0.02	0.725

Model fit measures: χ^2^_(227)_ = 424.728; CFI = 0.952; TLI = 0.909; RMSEA (95% CI) = 0.047 (0.040–0.054); SRMR = 0.046.

SN, subjective norm; PBC, perceived behavioral control.

Bold means statistically significant * means “Knowledge regarding the WHO dietary consumption recommendations”.

## 4 Discussion

This study examined the psychosocial and contextual factors affecting F&V consumption among adult Saudi women. It used the TPB and its extended model. Understanding these factors is important not only for addressing individual issues but also for promoting healthier eating habits in families. In Saudi Arabia, as in many Arab societies, women are key in managing food-related tasks, including meal planning, preparation, and serving. This makes them important in shaping the dietary habits and overall health of their families (32, 33).

The measurement model showed that Saudi women had very positive attitudes toward eating F&V, considering them healthy and beneficial. However, this positive attitude did not match the same level of PBC or SN, which were only moderately positive. This finding aligns with the results of Menozzi et al. (22). Notably, family expectations were rated higher than those of friends. This highlights the strong family-oriented of Saudi culture, where family relationships and expectations strongly influence personal choices and behaviors (34, 35). Despite these positive psychosocial factors, actual eating patterns showed a significant gap. Participants reported consuming an average of only 3.33 servings per day, meeting the recommended five servings on just 1–2 days each week. This result is consistent with earlier research studies that highlights low intake of F&V among the Saudi population (12, 13, 36). It emphasizes the need for focused efforts to turn positive attitudes into regular healthy eating habits.

The structural equation modeling based on the traditional TPB framework showed that intention and PBC were significant predictors of behavior, explaining 31% of the variance. This finding is consistent with earlier studies using the TPB in dietary contexts, where intention and PBC consistently appeared as strong factors influencing eating behavior (14, 22, 37, 38). This suggests that while Saudi women express intentions to increase their intake, translating intention into action remains constrained by actual or perceived barriers such as lack of availability, time constraints, or social influences (39, 40). In other words, while intention is a necessary driver for Saudi women to consume F&V, it is insufficient on its own to guarantee action, a phenomenon widely recognized in health psychology as the “intention-behavior gap” (41, 42). Importantly, intention was strongly predicted by all three TPB constructs, with PBC emerging as the strongest predictor. This aligns with the TPB literature, which consistently identifies PBC as a critical determinant of dietary behaviors, especially in contexts where structural or cultural barriers exist (14, 25, 43). As such, in the present study, women were not highly involved in food purchasing and preparation. Moreover, the positive correlations among attitude, SN, and PBC indicate synergistic influences: favorable attitudes are reinforced by social expectations and a sense of control, amplifying intentions.

Extending the TPB model improved explanatory power, with 78% of the variance in intention and 45% in behavior, supported by strong model fit indices. Notably, PBC remained the only significant predictor of behavior, while intention was not significant. This confirms the gap between intending and acting. This confirms the gap between intending and acting. While this gap is a well-documented phenomenon in health psychology (41, 42), its manifestation in this study requires context-specific interpretation. The finding suggests that for Saudi women, forming an intention to improve the family's diet is distinct from the ability to act on that intention. Some cultural and practical factors inherent to the Saudi context could explain this gap. Despite expressing strong intentions to eat more fruits and vegetables, many women reported limited involvement in purchasing and preparing them—only 35.6% always purchased vegetables, and 35.4% always cooked or prepared fruits and vegetables. Moreover, the preferences of other family members might restrict women's ability to ensure the fruit and vegetable intake they intend to achieve. Additionally, the time and effort required to prepare healthy meals from scratch—compared with the convenience of traditional dishes or readily available food delivery services—can further overwhelm intentions to make dietary improvements (44, 45).

In the extended model, SN and PBC continued to predict intention, while attitude was no longer significant. This change is an important finding, suggesting that in Saudi Arabia, collective expectations and perceived control can influence food-related behaviors more than individual preferences. This shift can be viewed from two angles. First, it likely reflects a cultural influence where strong family values, similar to those in other Gulf states (46, 47), cause social norms and practical control to take precedence over personal attitudes in making decisions (48). Second, it indicates a statistical mediation effect, where the new contextual variables took over the explanation previously attributed to attitude. The significant impact of knowledge of WHO recommendations (coeff. = 0.29, *p* = 0.000) and family meal frequency (coeff. = 0.20, *p* = 0.000) shows that these factors are fundamental in shaping and possibly mediating how positive attitudes translate into intention (49, 50).

These findings reveal strong intervention opportunities. Improving nutrition knowledge can strengthen positive attitudes at the core level, while encouraging regular family meals can create an environment that supports healthy eating habits and boosts self-efficacy, as shown in regional studies (46, 47). Moreover, the model shows that PBC is influenced by practical, contextual factors. The result that purchasing F&V and vegetables and maintaining a healthy diet were significant for PBC suggests that actual access and established healthy habits are essential in building women's confidence in meeting dietary guidelines (51). Thus, women who purchase more and follow a healthy diet likely develop higher self-efficacy, which directly enhances their PBC. This emphasizes that interventions must extend beyond education. They should actively improve access and develop practical skills, which would empower women's autonomy and control over their family's food choices.

## 5 Limitations

This research has important strengths, especially the use of an extended TPB framework. This framework allowed for examining various factors that influence F&V consumption among adult Saudi women. The sample included women from different age groups and geographic areas across Saudi Arabia, which broadens the findings. However, recruitment relied on online snowball sampling, which likely led to an overrepresentation of younger and more educated women. This sampling bias may limit the generalizability of the findings to the broader population of Saudi women, particularly those with lower educational attainment or limited internet access. However, some limitations should be noted. The cross-sectional design of the study makes it difficult to determine cause-and-effect relationships between the factors and dietary behavior (52). Also, relying on self-reported measures could introduce recall or social desirability bias (53). In addition, behavioral outcomes were assessed through self-reported fruit and vegetable consumption over a single week, which may overestimate actual adherence due to recall limitations. Future studies are encouraged to employ more objective and extended assessment methods, such as multiple 24-h dietary recalls, food diaries, or digital dietary tracking tools. Furthermore, relatively high correlations were observed among Attitude, Subjective Norm (SN), and Perceived Behavioral Control (PBC) (*r* ≈ 0.5–0.6), suggesting potential collinearity or limited discriminant validity among these constructs. Although this study did not calculate the Average Variance Extracted (AVE) or apply the Fornell–Larcker criterion, future research should include such analyses to formally assess construct validity and ensure that each TPB construct is conceptually and statistically distinct. To improve future evidence, researchers should consider longitudinal designs and objective assessment tools to strengthen causal inferences and measurement accuracy.

## 6 Conclusion

This study indicates that adult Saudi women demonstrated favorable attitudes toward F&V consumption; however, a gap between their intention and behavior was noticed. The core TPB model confirmed that intention and, more importantly, PBC were the main predictors of F&V consumption. The extended model demonstrated that PBC was the only direct predictor of behavior, overshadowing the effect of intention and further emphasizing the presence of the intention-behavior gap. Knowledge of recommendations, frequency of family meals, involvement in F&V purchasing, and overall healthy eating habits were viewed as significant determinants of TPB constructs, particularly attitudes and PBC. It is recommended to create family-based nutrition programs that utilize the influence of home practices. Integrating nutrition education that focuses on practical meal planning and preparation is also important. Empowering women as agents of dietary change within their families could help close the gap between intention and behavior. This approach can promote lasting healthy eating habits in Saudi households. Concluding with practical implications, initiatives such as family-based nutrition programs, community awareness campaigns, and women-centered skill-building interventions could help translate positive intentions into sustained healthy eating behaviors, thereby supporting policy and educational efforts to reduce the intention–behavior gap.

## Data Availability

The raw data supporting the conclusions of this article will be made available by the authors, upon reasonable request.
