# Supplementary material for: Expanding the theory of planned behavior to predict fruits and vegetables consumption among a sample of women in Saudi Arabia
Source: Front Public Health. 2025 Dec 4;13:1720598. doi: 10.3389/fpubh.2025.1720598 (PMC12713309; doi:10.3389/fpubh.2025.1720598)
Supplement: Supplementary file 1 [file Table_1.docx]

**Supplementary Table:** **Constructs and Cronbach's alpha**

| **Construct items** | **Alpha** |
| --- | --- |
| **Attitude**  Eating five servings of vegetables and fruits per day next week is:  Bad/good  Not pleasant/pleasant  Unhealthy/healthy  Indigestible/digestible | 0.76 |
| **Subjective Norm**  My family expects me to eat five servings of vegetables and fruits per day next week  My friends expect me to eat five servings of vegetables and fruits per day next week | 0.85 |
| **Perceived Behavioral Control**  I think that eating five servings of vegetables and fruits per day next week is possible  I am sure I can eat five servings of vegetables and fruits per day next week | 0.82 |
| **Intention**  I intend to eat five servings of vegetables and fruits per day next week  I am sure to eat five servings of vegetables and fruits per day next week  My aim is to eat five servings of vegetables and fruits per day next week | 0.92 |
| **Behavior**  Number of servings daily last week  Frequency of consumption | 0.72 |
